# Translational Research in Complementary and Alternative Medicine 2014

**DOI:** 10.1155/2015/427508

**Published:** 2015-01-19

**Authors:** Wei Jia, Aiping Lu, Kelvin Chan, Mats G. Gustafsson, Ping Liu

**Affiliations:** ^1^Center for Translational Medicine, Shanghai Jiao Tong University Affiliated Sixth People's Hospital, Shanghai 200233, China; ^2^Cancer Epidemiology, University of Hawaii Cancer Center, Honolulu, HI 96813, USA; ^3^School of Chinese Medicine, Hong Kong Baptist University, Kowloon Tong, Hong Kong; ^4^Faculty of Pharmacy, The University of Sydney, NSW 2006, Australia; ^5^The National Institute of Complementary Medicine, School of Science & Health, University of Western Sydney, NSW 2791, Australia; ^6^Cancer Pharmacology and Computational Medicine, Department of Medical Sciences, Uppsala University, Uppsala Academic Hospital, 751 85 Uppsala, Sweden; ^7^Key Laboratory of Liver and Kidney Diseases (Ministry of Education), E-institute of Shanghai Municipal Education Commission, Institute of Liver Diseases, Shuguang Hospital Affiliated to Shanghai University of Traditional Chinese Medicine, Shanghai 201203, China

Complementary and alternative medicine (CAM) has often been regarded as a practical, holistic, and personalized medical approach. However, the CAM research community in the past two decades has witnessed a huge disconnection between clinical studies and preclinical studies including authentication, quality control, pharmacology, and toxicology of CAM agents. Meanwhile, novel translational research approaches, including cutting-edge-omics technologies, new bioinformatics tools, novel imaging modalities, well-designed clinical trial metrics, protocols, and outcome measures, are still lacking in the CAM research. In this special issue, we aim to promote research that can translate from bench to bedside in CAM diagnosis and treatments.

This issue contains seven papers, where two papers reported novel approaches to human metabolic disease diagnosis using metabolomics technology. X. Wang et al. reported a novel syndrome differentiation strategy investigated in a clinical study. A group of liver cirrhosis patients (*n* = 63) who were classified into two TCM syndromes, “Liver-Kidney Yin Deficiency” or “Dampness-Heat Internal Smoldering,” and healthy subjects (*n* = 31) were recruited, and a combined gas chromatography, as well as liquid chromatography mass spectrometry, was used to profile the urine samples of the study participants. The results underscored several key urinary metabolite markers, including glycoursodeoxycholate, cortolone-3-glucuronide, and L-aspartyl-4-phosphate, that can readily differentiate between the two TCM syndromes. T. Wu et al. applied a mass spectrometry-based metabolomics approach to characterize the distinct alterations of serum metabolites among diabetes patients who can be classified into “excess” and “deficiency” TCM syndromes. The results suggest that patients with the excess syndrome have more oxidative stress than the deficiency syndrome, highlighting a novel diabetic patient subtyping method using a metabolomics approach.

Dr. G. H. Seol's group reported an interesting clinical trial to investigate the effects of inhalation of the essential oil of* Citrus aurantium L. var. amara* (neroli oil) on menopausal symptoms, stress, and estrogen in postmenopausal women. Sixty-three healthy postmenopausal women were randomized to inhale 0.1% or 0.5% neroli oil (v/v in almond oil) or almond oil (control) for 5 minutes twice daily for 5 days. They report that systolic blood pressure and diastolic blood pressure were significantly lower among participants inhaling neroli oil than the control group, suggesting that neroli oil may have potential as an effective intervention to reduce stress and improve the endocrine system.

J. Qi et al. evaluated the therapeutic effect and mechanisms of a traditional Chinese medicine, Apocynum Tablet (AT), on cardiac hypertrophy in a mouse model of cardiac hypertrophy. AT is formulated mainly with* Apocynum*,* Chrysanthemum*, and* Fangchi* and widely used in China to treat patients with hypertension. Their experimental data provided evidence that AT inhibits cardiac hypertrophy from pressure overload and thus explained a possible mechanism of the effective AT treatment in patients with cardiac hypertrophy. The authors also suggested that selective ERK1/2 and AKT modulation for cardioprotection as possible therapeutic targeting may be feasible.

In the paper entitled “*The traditional kampo medicine tokishakuyakusan increases ocular blood flow in healthy subjects*,” S. Takayama et al. reported a clinical study with healthy volunteers to examine the effects of oral administration of kampo medical formulas on ocular blood flow. A crossover protocol was used to randomly administer 5 grams of one of the 4 kampo medical formulas to 13 healthy subjects (mean age: 37.3 ± 12.3 years). Laser speckle flowgraphy was used and blood pressure and intraocular pressure were also recorded in the study. The authors concluded that one of the kampo formulas, tokishakuyakusan, can significantly increase ocular blood flow, at 30 to 60 minutes after administration, without affecting blood pressure or intraocular pressure in healthy subjects.

T. Kakegawa et al. conducted a translatome analysis to investigate the mechanisms and modes of action of three diarylheptanoids isolated from a medicinal plant,* Alpinia officinarum*. The authors comprehensively identified the polysome-associated mRNAs in a human B lymphoblastoid cell line and examined changes to the mRNA profile caused by each of three* A. officinarum* diarylheptanoids. The microarray-based translatome analysis was able to reveal the inhibitory effects on proinflammatory mediators and cytotoxic and antiviral activity of plant bioactives, highlighting the application of omics technologies in advancing our understanding of molecular effects of CAM agents.

In the paper entitled “*A review of botanical characteristics, traditional usage, chemical components, pharmacological activities, and safety of Pereskia bleo (kunth) DC*” S. Zareisedehizadeh et al. provided an up-to-date and comprehensive review of the botanical characteristics, traditional usage, phytochemistry, pharmacological activities, and safety of* P. bleo*. The review highlighted the association between the traditional usage of the plant and the anticancer, antibacterial, and antinociceptive effects reported in different studies.

In summary, these 7 papers represent exciting CAM research activities with translational strategies embedded in design and context. The articles will help readers to follow translational research in CAM with a wide range of topics, from clinical trials to omics technologies and bioinformatics that will collectively contribute to an improved understanding of mechanisms and pharmacology of the CAM treatments.

